# Exceptional preservation of a Cretaceous intestine provides a glimpse of the early ecological diversity of spiny-rayed fishes (Acanthomorpha, Teleostei)

**DOI:** 10.1038/s41598-018-26744-3

**Published:** 2018-05-31

**Authors:** Donald Davesne, Pierre Gueriau, Didier B. Dutheil, Loïc Bertrand

**Affiliations:** 1 Department of Earth Sciences, University of Oxford, OX1 3AN Oxford, United Kingdom; 2 Centre de Recherche sur la Paléobiodiversité et les Paléoenvironnements, UMR7207 (CNRS-MNHN-Sorbonne Université), Muséum national d’Histoire naturelle, 75005 Paris, France; 3 IPANEMA, CNRS, ministère de la Culture, UVSQ, USR3461, Université Paris-Saclay, 91192 Gif-sur-Yvette, France; 4 Synchrotron SOLEIL, 91192 Gif-sur-Yvette, France

## Abstract

Acanthomorph teleosts (spiny-rayed fishes) account for approximately a third of extant vertebrate species. They appeared during the Late Cretaceous and have been a major component of aquatic biodiversity since the early Cenozoic. They occupy today most trophic levels and ecological niches in aquatic environments, however very little is known about those that were adopted by the earliest representatives of the group. Here, we report on an exceptional glimpse into the ecological diversity of early spiny-rayed fishes provided by the unusual preservation of a newly discovered specimen of the freshwater acanthomorph *Spinocaudichthys* from the Upper Cretaceous of Morocco. A combination of major-to-trace elemental mapping methods reveals that the gross morphology of the specimen’s intestine has been remarkably preserved owing to the rapid mineralization of iron hydroxides around it. Differing with the typically short and straight intestinal tract of carnivorous teleosts, the intestine in *Spinocaudichthys* is long and highly convoluted, indicating a probable herbivorous diet. Acanthomorphs would therefore have conquered various ecological niches in their early evolutionary history, prior to their subsequent phylogenetic diversification in both marine and freshwater environments that followed the K-Pg extinction event.

## Introduction

Spiny-rayed fishes of the clade Acanthomorpha are a major component of modern aquatic ecosystems. They account for approximately a third of extant vertebrate species, existing in every aquatic environments and at every trophic level^1,2^. They are predominantly found in oceans today and their early evolutionary history is known from many well-preserved fossil taxa found in marine outcrops of the Cenomanian (early Late Cretaceous) that document the appearance in the fossil record of most major modern acanthomorph groups^3,4^. Despite this relatively good early fossil record, we have little information on the ecology of the earliest acanthomorphs before their phylogenetic and ecological diversification that probably occurred around the K-Pg extinction event^4,5^. While modern acanthomorphs are also highly diversified in freshwater environments with taxa such as trout-perches (Percopsiformes), labyrinth fishes (Anabantiformes), killifishes (Cyprinodontiformes), cichlids (Cichlidae) and “true” perches (Percidae), their fossil record in continental outcrops is much scarcer than in marine sediments (presumably due to the lower fossilisation potential of continental deposits). Indeed, the only freshwater acanthomorph known by Cretaceous articulated fossils is *Spinocaudichthys oumtkoutensis* Filleul & Dutheil 2001, from the Cenomanian Jbel Oum Tkout Lagerstätte (OT1) of South-Eastern Morocco^6^.

**Figure 1:**
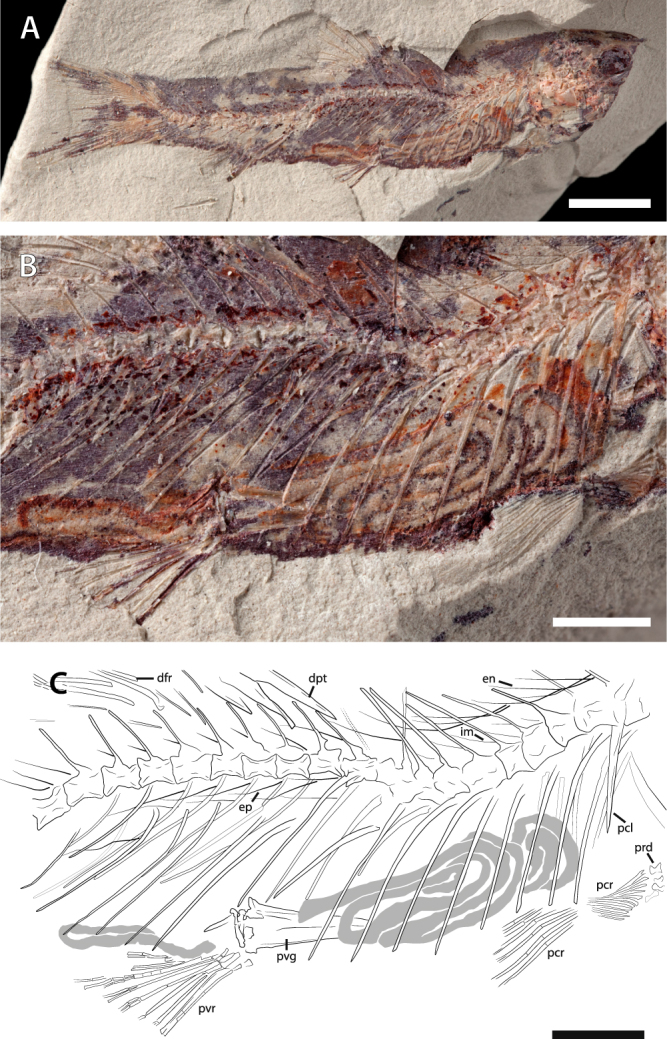
Exceptional preservation of the intestine in a specimen of the early spiny-rayed fish *Spinocaudichthys*. (**A**) Optical photograph of MHNM-KK-OT 09a; (**B**) Close-up of the abdominal region; (**C**) Interpretative drawing of the abdominal region. The intestine is highlighted in grey. Abbreviations: dfr, dorsal-fin rays; dpt, dorsal-fin pterygiophores; en, epineurals; ep, epipleurals; im, unidentified intermusculars (possibly epineurals or epipleurals); pcl, postcleithrum; pcr, pectoral-fin rays; prd, pectoral-fin radials; pvg, pelvic girdle; pvr, pelvic-fin rays. Scale bars, 5 mm (**A**) 2 mm (**B**,**C**).

A significant proportion of fossils from the OT1 Lagerstätte displays an exceptional preservation of soft tissues, with finely mineralized muscles showing fibre striation under scanning electron microscopy^7^. Here we report the preservation of an intestinal tract in a new specimen of *Spinocaudichthys* (Fig. 1). We describe the chemistry and provide insights into the preservation mechanism of the specimen based on a combination of synchrotron micro X-ray fluorescence major-to-trace elemental mapping and of scanning electron microscopy coupled to energy-dispersive X-ray spectroscopy (SEM-EDX). Its exceptional preservation allows drawing an integrative reconstruction of the palaeoecology of *Spinocaudichthys*, as well as of the taphonomy and burial environment of the fossil specimen. This work therefore offers an unexpected window into the palaeoecology of the earliest known freshwater acanthomorph representative, providing valuable insight on the ecological diversity of the group in the earliest steps of their successful evolutionary history.

## Results

### Systematic palaeontology

Teleostei Müller, 1845.

Acanthomorpha Rosen, 1973.

*Spinocaudichthys* Filleul and Dutheil, 2001.

#### Emended diagnosis

Elongated acanthomorph fish which differs from other acanthomorphs by the following combination of primitive and derived character states: 32 to 46 vertebrae; epineurals and epipleurals; no ossified supraneural; dorsal fin inserting posterior to neural spine 10 to 12; four spines and eight soft rays in dorsal fin; three spines and five soft rays in anal fin; ten dorsal and eight ventral procurrent spines in caudal fin; 20 principal rays in caudal fin; long neural spine of the second preural centrum; two ural centra unfused to the hypurals; two epurals; six unfused hypurals; two unfused uroneurals; free pelvic girdle inserted posterior to the postcleithra; pectoral fins low on the flanks; no spine in the pelvic and pectoral fins.

*Spinocaudichthys oumtkoutensis* Filleul and Dutheil, 2001.

#### Holotype

**Figure 2:**
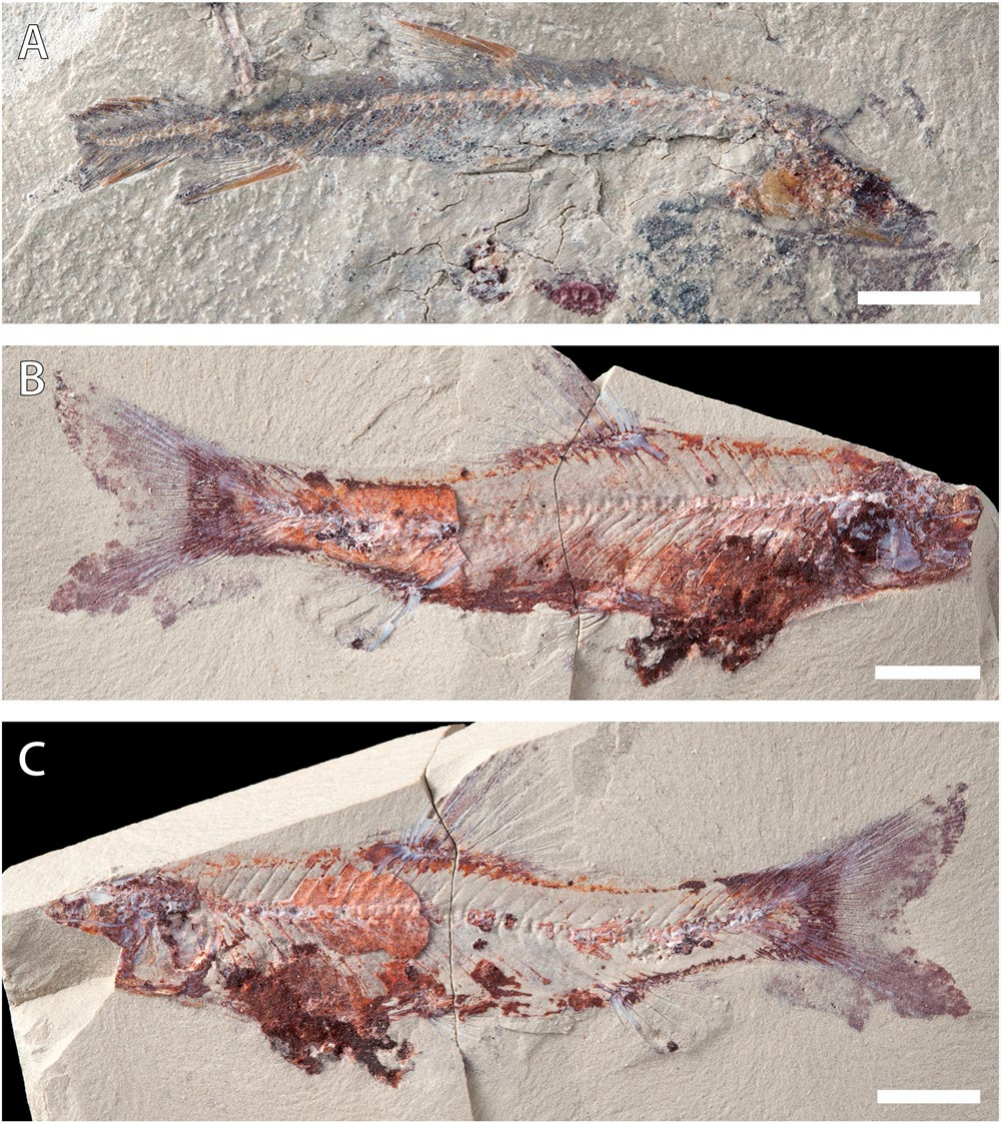
Additional specimens of *Spinocaudichthys*, including newly discovered specimens. (**A**) *S*. *oumtkoutensis*, holotype, POI-SGM 20a; (**B**) *Spinocaudichthys* sp., MHNM-KK-OT 12a; (**C**) *Spinocaudichthys* sp., MHNM-KK-OT 12b, counterpart. Scale bars, 5 mm.

POI-SGM 20a-b (Fig. 2A).

*Spinocaudichthys* sp.

#### Referred specimens

**Figure 3:**
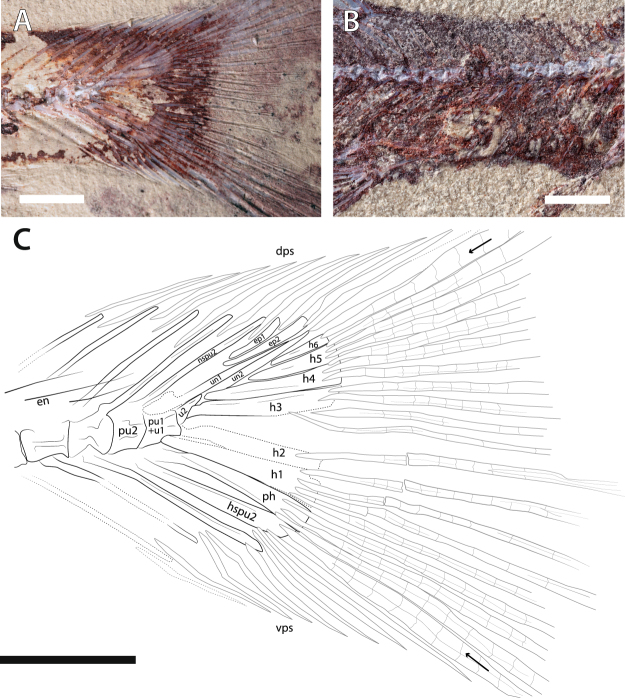
Close-ups of newly discovered *Spinocaudichthys* specimens. (**A**) *Spinocaudichthys* sp., MHNM-KK-OT 12b, close-up of the caudal skeleton; (**B**) *Spinocaudichthys* sp., MNHN-KK-OT 11, close-up of the partially preserved intestine; (**A**) MHNM-KK-OT 12b, interpretative drawing of the caudal skeleton. Black arrows signal the first and last principal caudal-fin rays. Abbreviations: dps, dorsal procurrent spines; en, epineural bones; ep1-2, epurals; h1-6, hypurals; hspu2, haemal spine of the second preural vertebra; nspu2, neural spine of the second preural vertebra; ph, parhypural; pu1, first ural vertebra; pu2, second preural vertebra; u1, first ural centrum; u2, second ural centrum; un1-2, uroneurals; vps, ventral procurrent spines. Scale bars, 2 mm.

MHNM-KK-OT 09a-b (Fig. 1), MHNM-KK-OT 11, MHNM-KK-OT 12a-b (Figs 2 and 3), new specimens discovered during a 2012 field mission to the Jbel Oum Tkout Lagerstätte. MHNM-KK-OT 09 displays an exceptional preservation of most of the intestine (Fig. 1).

### Description of the material

The *Spinocaudichthys* specimens described here (Figs 1–3) have been collected in 2012, subsequently to the original description. They can be referred as *Spinocaudichthys* based on shared characters with the holotype in the postcranium, notably in the caudal skeleton (number of caudal-fin rays and procurrent spines, same structure of the caudal vertebrae and associated bones) and in the meristic counts of fin rays (number of dorsal-, anal- and pelvic-fin rays and spines). However, the material referred here differs from the holotype (Fig. 2A) in its vertebral count, that of the holotype being unambiguously higher. Therefore, and without excluding intra-specific variation, we choose to adopt the conservative position of referring this new material as *Spinocaudichthys* sp., since it potentially corresponds to a new species. In this description we insist chiefly on the morphological features missing or incorrectly reported from the holotype. For a more in-depth account of the morphology, readers can refer to the original description^6^.

As in the holotype, preservation of the available material does not provide much information in the anatomy of the cephalic region. The cranial roof does not seem to bear a sagittal crest as in some early acanthomorphs: we interpret the ‘supraoccipital crest’ of the holotype as a displaced cranial roof bone. The parasphenoid is narrow and slightly sinusoidal in shape. In MHNM-KK-OT 12 (Fig. 2B,C), the anterior ceratohyal is visible; it is longer than deep and its anterior extremity seemingly does not form a condyle-like articulation with the (non-preserved) hypohyals. In the same specimen, the hyomandibula is partially preserved, apparently bearing a single articular head contacting the neurocranium. The opercular series is poorly preserved most of the time, but MHNM-KK-OT 12 shows a relatively complete preopercle noticeably angled at mid-length (Fig. 2B,C).

There is some variation in the vertebral counts. While the holotype has at least 45, the number of visible vertebrae in the new material ranges between 32 and 34, with the exact number uncertain due to poor preservation of the anterior-most portion of the vertebral column. As in the holotype, the vertebrae are longer than deep, except for the four to five anterior ones; they are constricted in the middle, and their neural spines are all similarly thin and angled posteriorly. In the abdominal vertebrae, we did not find any evidence of ventral parapophyses. Caudal vertebrae have haemal spines, approximately the same lengths as their neural spines, but stouter. Ribs are present up to vertebra 23–25 in MHNM-KK-OT 09, but to vertebra 31 in the holotype. They insert directly on the vertebral centra, in the absence of parapophyses. At least two intermuscular series are present. In MHNM-KK-OT 09, an almost complete epineural series is preserved, with the bones inserting from the first vertebra to the fourth preural centrum (Figs 1B,C and 3A,C). The anterior epineurals insert on the neural spines, while the posterior-most ones insert at the base of the corresponding neural arch or possibly lower. In the same specimen, the left and right epipleural series insert ventral to the centra of the 11^th^ or 12^th^ vertebra up to the fifth or sixth preural centrum. Finally, in this specimen the distal extremities of at least six more ossified intermusculars are visible (Fig. 1c), ranging from the fourth to the eighth vertebra. They could be interpreted as anterior epipleurals, ventrally displaced epineurals from the opposite side of the specimen, or as epicentrals. As the identity of these intermusculars is doubtful, we consider that two series, potentially three, are found in the taxon.

The dorsal fin consists in nine pterygiophores supporting four spines and eight segmented and dichotomous rays. No ossified supraneurals are preserved in our material. The three anterior pterygiophores are longer and broader than the gracile posterior ones. The first dorsal-fin pterygiophore inserts posterior to the tenth neural spine in our studied material, except in the holotype where it seems to insert posterior to the 12th neural spine. Each pterygiophore inserts in one consecutive interneural space, except for pterygiophores five and six, which share the same interneural space. Dorsal-fin spines are increasing in length, each being approximately twice as long as the preceding. All dorsal-fin soft rays seem to bifurcate distally. The anal fin consists in seven pterygiophores (the anterior two noticeably enlarged and in contact with each other throughout their length) supporting three spines of increasing length and five segmented rays. It inserts ten to twelve vertebrae behind the dorsal fin.

The caudal fin and skeleton are well-preserved in a number of specimens (Figs 2B,C and 3A,C), allowing to confirm most of the observations made on the type material, but also to reinterpret some. There are two discrete ural centra and the neural spine of the second preural centrum is long and narrow (Fig. 3A,C). It is unclear wether the first preural centrum bears an “incomplete” neural spine as described from the holotype: based on other material we propose that it could be interpreted as the enlarged base of the first uroneural. Only two epurals are present, inserting between the second preural neural spine and the first uroneural. The original publication described three separate uroneurals and five hypurals. However, our reevaluation of the material supports that what was interpreted as the third uroneural is actually the sixth hypural. Hence, there are two uroneurals and six hypurals (four dorsal and two ventral) in the caudal skeleton of our material (Fig. 3A,C). There are ten dorsal and eight ventral procurrent spines inserting anterior to the caudal-fin rays (eight dorsal spines were described from the holotype owing to incomplete preservation of the material). There are 20 principal caudal-fin rays, of which eleven are in the dorsal lobe and nine in the ventral lobe. The ventral-most ray inserts on the haemal spine of the second preural vertebra, while the dorsal-most inserts on the sixth hypural. The caudal fin is noticeably forked (Figs 1A, 2B,C and 3A).

The pectoral girdle is poorly preserved in our material. The postcleithrum is a straight rod slightly oriented posteriorly and which ventral extremity seemingly does not reach the ventral margin of the body. Pectoral fins insert low on the body. In the holotype, 13 rays are visible and at least 11 in MHNM-KK-OT 09 (Fig. 1C), in which four hourglass-shaped pectoral-fin radial are faintly preserved. There is no pectoral spine. The pelvic girdle inserts posteriorly, its anterior tip at the level of the tenth or eleventh vertebra and posterior to the ventral tip of the postcleithrum. It does not contact the pectoral girdle. The preservation is not sufficient to describe in details the structure of the narrow and elongate pelvic bones. In MHNM-KK-OT 09 (Fig. 1B,C), they bear prominent median processes and at least one posterior process is preserved. Their internal wings are narrow and seem to contact each other near their anterior tip. Up to eight pelvic-fin rays are preserved. The anterior-most, while thicker than the others, is segmented and bifurcated distally and is therefore not a pelvic spine.

In at least two specimens, portions of the intestine are preserved (Figs 1, 3B). More remarkably, the intestinal tract is almost complete in the specimen MHNM-KK-OT 09 (Fig. 1). It lies ventrally in the abdominal cavity, between the pectoral girdle and the anal fin like in extant teleosts. Preserved as a flat imprint, its morphology can be accurately described thanks to its white to pale yellow colour that stands out from bluish or reddish surrounding tissues. The intestine appears to be highly convoluted (Fig. 1B,C), with at least nine visible bends. We estimate its uncoiled length to be about 1.5 times the length of the animal. The anterior part of the gastrointestinal tract (oesophagus, stomach if any, and pyloric caeca) is seemingly not preserved. Preservation of the intestine interrupts at the level of the pelvic skeleton, resuming posteriorly. This posterior section might correspond to parts of the hindgut but the posterior-most portion (rectum and anal vent) appear to be missing.

### Chemical characterization of the intestinal tract

**Figure 4:**
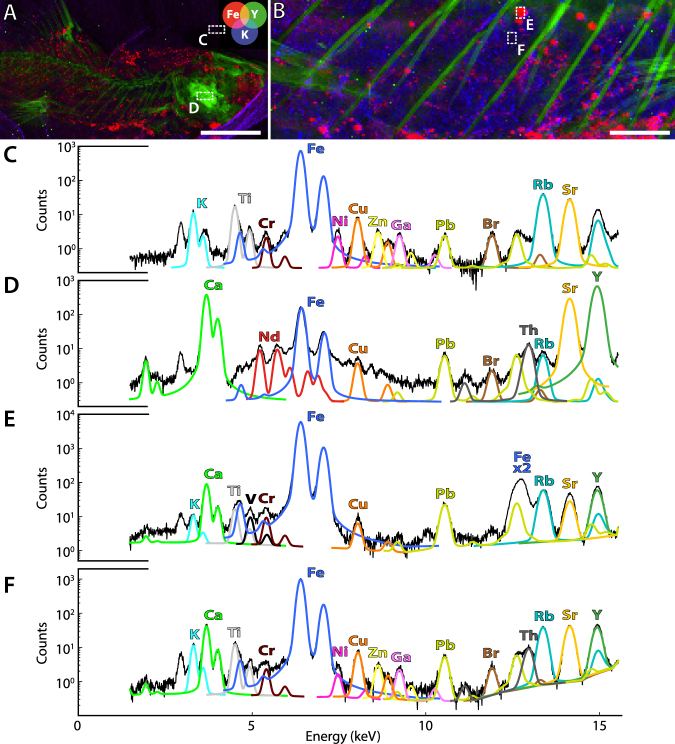
Synchrotron micro-X-ray fluorescence (µXRF) maps of major-to-trace elements of MHNM-KK-OT 09a. (**A**) Iron (red), yttrium (green) and potassium (blue) distributions of the anterior part of the specimen; (**B**) Distributions of the same elements at a greater lateral resolution of the intestinal tract, reconstructed from a full spectral decomposition of the XRF data (information depth typically 50 µm for Fe and K and 150 µm for Y). Acquisition parameters: 100 × 100 µm^2^ scan step, 28,951 pixels in (**A**); 30 × 30 µm^2^ scan step, 18,018 pixels in (**B**). Scale bars, 5 mm (**A**) 1 mm (**B**). (**C**–**F**) Mean XRF spectra and main elemental contributions from the box areas in (**A**) (mean spectra from 65 pixels) and (**B**) (mean spectra from 40 pixels), respectively. From top to bottom, spectra are respectively characteristic of the sedimentary matrix, a skull bone, an iron-rich grain and the intestine. Note that the vertical scale differs in (**E**).

**Figure 5:**
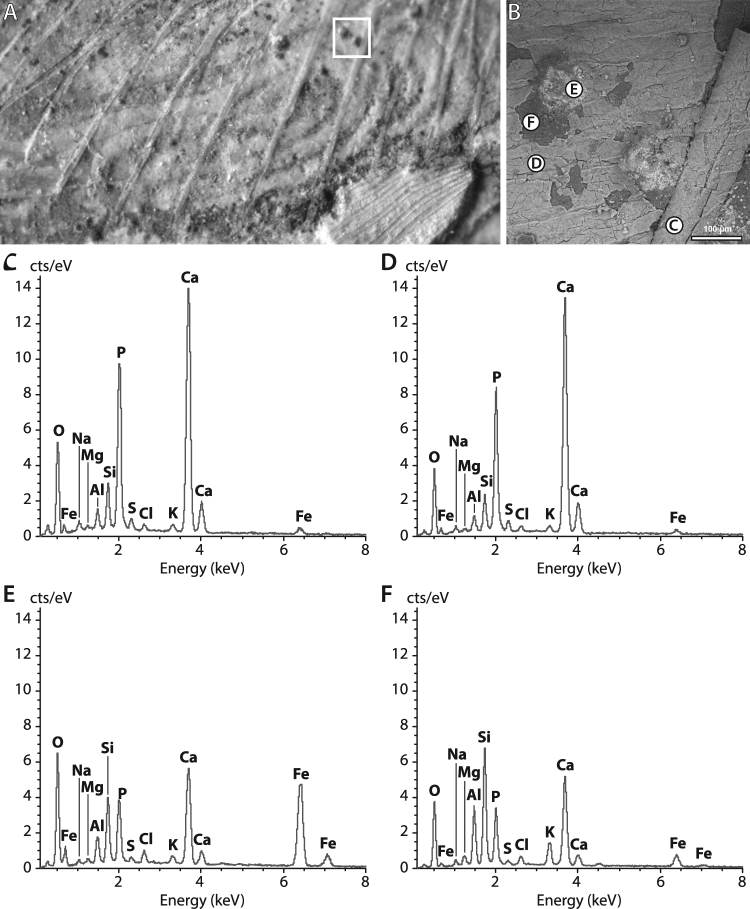
SEM image and energy-dispersive X-ray spectroscopy analysis of the intestine of *Spinocaudichthys*, MHNM-KK-OT 09a. (**A**) Optical photograph of the intestine; (**B**) SEM image (BSE mode) from the white box area in (**A**) showing part of an abdominal rib (right) and two iron-rich grains. Scale bar represents 100 µm; (**C**–**F**) Energy-dispersive X-ray spectra from areas indicated in (**B**), respectively characteristic of bone (**C**), the intestine (**D**), an iron-rich grain (**E**) and the sedimentary matrix underneath the intestine (**F**).

Despite quickly disappearing during decay^8^, digestive systems and intestines in particular are regularly the only portions of the soft internal anatomy preserved in fossil vertebrates, notably actinopterygians^9–11^. They are reported to be primarily mineralized, in three dimensions, through phosphatization. In order to understand the different mode of exceptional preservation of the intestinal tract in MHNM-KK-OT 09, and to uncover any potential biases likely to affect the robustness of further palaeobiological and palaeoecological reconstructions inferred from this fossil, we explored its chemical composition with a combination of major-to-trace elemental mapping methods: synchrotron-based micro X-ray fluorescence (µXRF) and energy-dispersive X-ray spectroscopy (SEM-EDX). Synchrotron µXRF mapping of major-to-trace elements at 100-µm scan step clearly highlights the skeleton from the sedimentary matrix and the soft tissues (Fig. 4A). Similar to other actinopterygians from the same locality^12^, the spatial distributions of calcium, strontium, yttrium (green overlay in Fig. 4A), several rare earth elements (REEs; in particular here neodymium, samarium, europium and erbium that display the most contrasted distributions), and thorium (see the mean X-ray fluorescence spectrum from the operculum in Fig. 4D) are essentially confined to skull bones, axial skeleton and fin rays. Soft tissues appear richer in iron (red overlay in the false colour elemental maps) and the clayey sedimentary matrix in potassium (blue overlay). A higher resolution mapping of the gut area (30-µm scan step; Fig. 4B) allows distinguishing the gross morphology of the intestinal tract, enriched in iron. X-ray fluorescence spectra collected on the intestine itself (i.e. within the iron outline; Fig. 4F) reveal a composition very similar to that of the sedimentary matrix (Fig. 4C) yet with increased calcium, yttrium and thorium content. Trace elements such as Y, lanthanides and Th are known to substitute for calcium in calcium phosphates such as apatite group minerals constituting fossil bones or mineralized soft-tissues, their ionic radii being very close to that of calcium^12^. SEM imaging coupled to induced X-ray emission spectroscopy (whose information depth, unlike for synchrotron XRF, is restricted to the very surface–a few µm) confirms the presence of a very thin layer of calcium phosphate (most probably apatite as observed in other fossils from the locality^13^) covering most of the specimen (including the intestinal tract) and similar in composition to bones (Fig. 5A–D). While the intestinal tract itself is only faintly mineralized in calcium phosphate, its morphology can be precisely described in the fossil owing to the continuous peripheral presence of polydisperse iron-rich grains (from a few to 50–150 µm in diameter as revealed by SEM-EDX; Figs 4B and 5A,B).

Most fossils from the OT1 Lagerstätte are covered by films made of iron-rich grains such as those drawing the outline of the intestine in MHNM-KK-OT 09. Using synchrotron transmission X-ray diffraction on another well-preserved actinopterygian from the locality, Gueriau *et al*.^13^ identified these phases as iron hydroxides. REE geochemistry at the site further reveals preservation of an early diagenetic signal with no secondary recrystallization^13^, therefore advocating for a burial origin of these iron oxides. Although the latter are common precipitates in oxygenated waters, the presence of fossil microbial mats at the site rather suggests that ferric hydroxide precipitation was induced by the metabolic activity of Fe-oxidizing bacteria, another well-known origin for iron oxide precipitation as a metabolic by-product^14^. This is also in line with the exquisite fossilization of muscles in most OT1 crustaceans and actinopterygians^7^, as microbial activity is assumed to allow the deposition at the sediment−water interface of the significant amount of dissolved phosphorus that is required for an extensive authigenic mineralization of the soft tissues in calcium phosphate minerals^15–17^.

Our in-depth (geo-)chemical characterization also highlights two potential taphonomic biases or limitations to consider: (1) the position of the iron-rich grains around the gut may imply that the latter appears slightly larger than it really was; (2) their size of 5–150 µm (low level of fidelity compared to fossilization through phosphatization that involves crystallites < 30 nm^16^) does not allow preserving histological details. Nevertheless, the rapid, most likely microbially-induced mineralization of iron hydroxides around the gut of MHNM-KK-OT 09 ensured the preservation of its gross morphology, which is strongly related to food habits and diet in vertebrates, providing a unique glimpse into the ecology of early acanthomorphs.

## Discussion

### Systematic position of *Spinocaudichthys*

**Figure 6:**
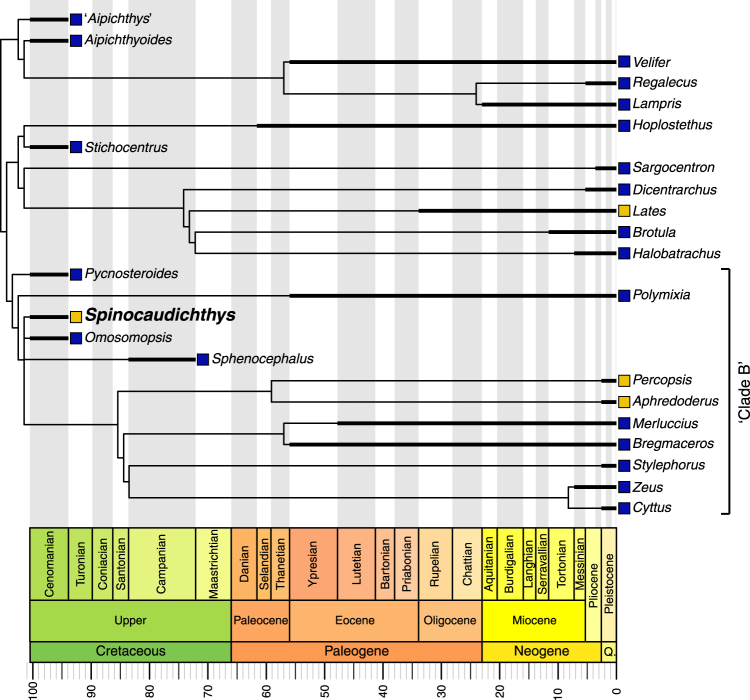
Phylogenetic analysis of acanthomorph teleosts, plotted on stratigraphy. Strict consensus of six parsimonious trees. The matrix is adapted from Davesne *et al*.^22^. Stratigraphic ranges of terminal taxa are indicated by thick lines, with internodes positioned at the age of the oldest known fossil when relevant. Yellow squares denote freshwater taxa, blue squares marine taxa. Non-acanthomorph outgroups are not figured. The figure was generated using the *strap* package^65^ in R.

Due to the presence of spines on the dorsal and anal fins in *Spinocaudichthys*, it was attributed to Acanthomorpha in the original description, but with an uncertain position within the clade due to a unique combination of character states^6^. However, the recently renewed phylogenetic framework for deep acanthomorph intrarelationships that is simultaneously supported by molecular^5,18–20^ and morphological data^21,22^ provides new insight on that matter. Moreover, the newly discovered *Spinocaudichthys* sp. material (Figs 1–3) provides new characters contributing to unravel its systematic position. We confirmed the attribution of *Spinocaudichthys* to acanthomorphs and resolved its phylogenetic position within the clade by adding it to the morphological character matrix of Davesne *et al*.^22^ (Fig. 6).

The principal acanthomorph synapomorphy that is visible in *Spinocaudichthys* specimens is the presence of spines in the dorsal and anal fins^6,22,23^. This character can be somewhat misleading in fossil taxa, because dorsal-fin spines are also present in other teleosts such as a few Cyprinidae and most Siluriformes^24^. However, the spines in *Spinocaudichthys* are indistinguishable from typical acanthomorph spines: several spines are found in both the dorsal and anal fin (but not on the pectoral fins), they increase in length posteriorly, have a pointed extremity and are not serrated (Figs 1, 2). This distribution of spines is not found elsewhere in teleosts. Moreover, none of the synapomorphies that would support an attribution to either siluriforms or cyprinids (such as the Weberian apparatus or the second hypural bone fused to the ural centra^23^) are found in *Spinocaudichthys*.

*Spinocaudichthys* combines derived character states of certain specific acanthomorph subclades (see below) with features that are plesiomorphic for acanthomorphs as a whole (e.g., two series of intermuscular bones, two distinct ural centra) but are nevertheless present in most known Cenomanian acanthomorphs and in some extant taxa such as *Polymixia*^3,22^. Even its characters that seem to diverge from the early acanthomorph bauplan (e.g., elongate body, pectoral fins inserting low on the body, posteriorly located pelvic fins) are also found in some modern taxa, for example the freshwater *Percopsis*. It then appears than the combination of morphological osteological characters displayed by the multiple *Spinocaudichthys* specimens at our disposal matches either that of some Late Cretaceous acanthomorphs or of some modern representatives, but could not be found in any other teleost group.

Scoring these characters in a morphological matrix (see ‘Phylogenetic analysis’ below) allow us to assign *Spinocaudichthys* a well-supported position in the acanthomorph tree (Fig. 6). In the strict consensus tree recovered by our phylogenetic analysis, *Spinocaudichthys* is included within “clade B” of Davesne *et al*.^22^ along with *Omosomopsis*, a marine acanthomorph also found in the Cenomanian of Morocco^25,26^ (Fig. 6). “Clade B” also includes the extant marine Polymixiiformes, Gadiformes and Zeiformes along with the freshwater Percopsiformes^5,21,22^. The inclusion of *Spinocaudichthys* in this clade is supported notably by its full neural spine on the second preural vertebra^22^. *Spinocaudichthys* is also more closely related to the [Percopsiformes + Gadiformes + Zeiformes] clade than to *Polymixia* because it has two epural bones instead of three, but does not belong to this clade due to the plesiomorphic state of some of its characters (e.g., it retains ossified epipleural bones and a second ural centrum separated from the hypurals)^22^. In addition to *Spinocaudichthys*, “clade B” includes several Late Cretaceous marine fossil taxa, including the extinct sphenocephalids (Fig. 6) and the earliest known zeiform^22,27–29^. It is noteworthy that *Spinocaudichthys* shares its freshwater habitat, relative positions of paired fins and elongated body shape with modern Percopsiformes, but the two are not recovered as sister taxa in our analysis.

### Diet reconstruction

Morphology of the alimentary canal in vertebrates is strongly related to food habits and diet. Convolutions of the intestine in teleosts, as observed in *Spinocaudichthys* sp., allow the elongation of the digestive tract within a limited volume and increases the surface area for nutrient absorption^30^. Relative intestinal length therefore reflects the nature of the dominant food items consumed by the animal, and herbivorous taxa (or omnivores including plants as part of their diet) generally show a significantly higher relative intestine length compared with carnivores, due to plant-based meals being more difficult to digest^30–32^. This relationship has been observed multiple times in teleosts at different phylogenetic scales^33–35^, more specific examples being Neotropical freshwater taxa^36,37^, marine acanthurids^38^ and stichaeids^39^ or African lake cichlids^40^. In MHNM-KK-OT 09, the preservation of a very long intestine compared with body size (~1.5 times longer than body length) with a high number of convolutions (at least 6–8 convolutions; Fig. 1B,C) then hints at a probable herbivorous diet for *Spinocaudichthys* sp. In this case, feeding ecology could be linked with habitat and with a greater availability of plant nutrients in lakes or rivers due to their proximity with terrestrial ecosystems. The transition from carnivory to herbivory is proposed to provide some adaptive advantages to freshwater species, such as an easier access to nutrients, a facilitation to conquer habitats with low prey density and a reduced exposure to disease transmission^41^.

### Early ecological diversity of spiny-rayed fishes

According to most studies, acanthomorph teleosts underwent a rapid morphological and phylogenetic diversification in the Palaeogene, seemingly driven by the vacancy of numerous morpho-functional niches left vacant after the end-Cretaceous mass extinction^4,42–44^. Conversely, early Late Cretaceous acanthomorphs were much more restricted in their morphospace, most of them showing a similar range of morphologies with small sizes and laterally compressed bodies with high depth ratios^4^. With its elongate body, *Spinocaudichthys* then appears as an outlier within the morphological disparity of Cenomanian acanthomorphs.

The other fossil deposits that yielded Late Cretaceous acanthomorphs are all marine^3,26,29,45–49^, with the exception of a few isolated bones attributed to Percopsiformes from the Campanian of Alberta and the Maastrichtian of Montana^50,51^, and of specimens preserved in possibly freshwater intertrappean cherts from the end-Maastrichtian of India^52^. Therefore, the Cenomanian *Spinocaudichthys* is unambiguously the oldest known, and potentially the only articulated Cretaceous record, of freshwater acanthomorphs (Fig. 6). The scarcity of freshwater acanthomorphs in the Cretaceous (although potentially explained by the rarer freshwater localities from this period) parallels the phylogenetic repartition of modern freshwater taxa in the acanthomorph tree, with a reconstructed marine origin for the group as a whole, followed by multiple secondary conquests of freshwater environments^53^.

Our reconstruction of an herbivorous diet for *Spinocaudichthys* sp. also contributes to the uniqueness of this taxon amongst coeval taxa. Other early Late Cretaceous acanthomorphs include close relatives to modern groups Lampridiformes^54,55^, Polymixiiformes^29^ and Beryciformes^45,46^ (Fig. 6). While feeding ecology of these extinct taxa cannot be inferred directly due to the lack of fossils with preserved soft tissues, modern representatives of these groups are all carnivores. For example, the freshwater Percopsiformes, superficially similar to *Spinocaudichthys* in their gross morphology, show a much less tortuous and shorter intestine reflecting a largely carnivorous diet^56^. Herbivory in acanthomorphs is then probably a secondary feature, exemplified by its post-Cretaceous expansion in marine reef-associated faunas^57^.

In its gross morphology, living environment and putative feeding ecology *Spinocaudichthys* is then markedly different from every other known Late Cretaceous acanthomorph. The freshwater ecology of the taxon indicates that some acanthomorph lineages had already conquered continental environments in the Cenomanian, while its inferred herbivorous diet shows that the trophic ecology of the group was much more diverse at this period than previously thought. The description of this new specimen then illustrates the conquest of various niches in the early evolutionary history of acanthomorphs, suggesting that this mega-diverse vertebrate clade underwent an ecological radiation earlier than previously thought.

## Material and Methods

### Geological background

The Jbel Oum Tkout locality (OT1) crops out within unit II of the Kem Kem beds, early Cenomanian (Late Cretaceous) in age^58^. The OT1 Lagerstätte yielded numerous fossil remains including a rich flora of gymnosperms and angiosperms, as well as unionoid bivalves, aquatic insects, isopod and decapod malacostracans, hybodont elasmobranchs and actinopterygians^6,7,59–61^. Fossils have been recovered from five centimeters-thick successive grey illitic layers exhibiting mudcraks. The absence of marine organisms and the presence of mudcracks, unionoids and larvae of odonatopterans and ephemeropterans points to a low-energy seasonally dried freshwater habitat comparable to a small lake, pool or oxbow lake^7,61^.

### Fossil material

The specimen studied herein (MHNM-KK-OT 09, Fig. 1) was collected in a November 2012 field trip that yielded at least 50 other specimens provisionally referred to *Spinocaudichthys* sp., of various qualities of preservation. A few (e.g., MHNM-KK-OT 11, Fig. 3B) show portions of the intestine fossilised, but MHNM-KK-OT 09 is the only one in which the intestine is preserved almost completely and *in situ*. This specimen has been prepared mechanically to unravel the posterior portion of its body, notably the caudal skeleton. All collected material belongs to the Musée d’Histoire naturelle de Marrakech (Morocco; MHNM), but is currently housed at the Muséum national d’Histoire naturelle (Paris, France; MNHN) for study, within an agreement between both museums.

### Phylogenetic analysis

*Spinocaudichthys* was coded (from both the *S*. *oumtkoutensis* and *S*. sp. material) for the 66 morphological characters of the Davesne *et al*.^22^ matrix (Supplementary Information). The matrix was submitted to parsimony analyses using PAUP* 4.0a^62^, rooting the tree on the aulopiform *Synodus*. Every character was treated as unordered, and multi-state taxa were treated as uncertainties. We performed a heuristic search with a random addition sequence and the “TBR” branch-swapping algorithm (10000 replicates, 10 trees held at each step). The analysis yielded six parsimonious trees with a length of 197 steps, a consistency index (CI) of 0.401 and a retention index (RI) of 0.683.

### Major-to-trace elemental mapping and spectroscopy

The chemical composition of the fossil was investigated using synchrotron-based micro X-ray fluorescence (µXRF) mapping, as well as SEM imaging coupled to energy-dispersive X-ray spectroscopy. µXRF maps were collected at the DiffAbs beamline of the SOLEIL synchrotron (Gif-sur-Yvette, France)^63^, using an excitation energy of 17.2 keV, selected for excitation of K-lines from phosphorus to yttrium and L-lines from cadmium to lead. The incoming X-ray beam was collimated by 2 bendable mirrors, monochromatized using a Si(111) double-crystal monochromator (energy resolution: 0.7 eV) and focused using Kirkpatrick-Baez mirrors down to a spot size of 11 × 7 µm^2^ (H × V, full width at half maximum). The sample was mounted on a xyz scanner stage, allowing ± 12 mm movements with micrometre accuracy. The sample was oriented at 45° to the incident beam and at 45° to the XRF detector, a mono element silicon drift detector (SDD, Vortex EX, total active area: 100 mm^2^) placed in the horizontal plane. Counting time per pixel was set to 500 ms to attain good statistics on trace elements. All the elemental distributions presented herein have been reconstructed from full spectral decomposition performed with the PyMCA data-analysis software^64^ using a batch-fitting procedure, polynomial baseline subtraction, and assuming a Pseudo-Voigt peak shape.

SEM imaging coupled to energy-dispersive X-ray spectroscopy was performed at the MNHN microscopy platform in backscattered electron (BSE) mode using a Tescan SEM (VEGA II LSU, low vacuum: 10–48 Pa, 20 kV) coupled to a SDD X-ray detector (SD^3^, Bruker).

## Electronic supplementary material

Supplementary Information
