# Supplementary material for: Exceptional preservation of a Cretaceous intestine provides a glimpse of the early ecological diversity of spiny-rayed fishes (Acanthomorpha, Teleostei)
Source: Sci Rep. 2018 May 31;8:8509. doi: 10.1038/s41598-018-26744-3 (PMC5981375; doi:10.1038/s41598-018-26744-3)
Supplement: Supplementary file 1 — Supplementary Information [file 41598_2018_26744_MOESM1_ESM.docx]

**Exceptional preservation of a Cretaceous intestine provides a glimpse of the early ecological diversity of spiny-rayed fishes (Acanthomorpha, Teleostei)**

**-**

**Supplementary Information**

**Authors:** Donald Davesne^1,2,*^, Pierre Gueriau^2,3,4,*^, Didier B. Dutheil^2^ and Loïc Bertrand^3,4^

^1^ Department of Earth Sciences, University of Oxford, OX1 3AN Oxford, United Kingdom.

^2^ Centre de Recherche sur la Paléobiodiversité et les Paléoenvironnements, UMR7207 (CNRS-MNHN-UPMC), Muséum national d’Histoire naturelle, 75005 Paris, France.

^3^ IPANEMA, CNRS, ministère de la Culture, UVSQ, USR3461, Université Paris-Saclay, 91192 Gif-sur-Yvette, France.

^4^ Synchrotron SOLEIL, 91192 Gif-sur-Yvette, France.

*These authors contributed equally to this work. Correspondence should be addressed to D.D. (email: donald.davesne@earth.ox.ac.uk) or P.G. (email: pierre.gueriau@synchrotron-soleil.fr).

**Taxon-by-character matrix used in the phylogenetic analysis.**

The following taxon-by-character matrix is adapted from Davesne *et al.* (2016). It adds the taxon †*Spinocaudichthys*, from the Late Cretaceous of Morocco, to the matrix.

For the full list of characters and character states, see the Supplementary Material of Davesne *et al.* (2016) at:

https://www.frontiersin.org/articles/10.3389/fevo.2016.00129/full#supplementary-material

Missing character states are coded as '?'. Inapplicable character states are coded as '-'.

**References**:

Davesne, D., Gallut, C. Barriel, V., Janvier, P., Lecointre, G., Otero, O. (2016) The phylogenetic intrarelationships of spiny-rayed fishes (Acanthomorpha, Teleostei, Actinopterygii): fossil taxa increase the congruence of morphology with molecular data. *Frontiers in Ecology and Evolution* 4: 129.

|  | **1** | **2** | **3** | **4** | **5** | **6** | **7** | **8** | **9** | **10** | **11** | **12** | **13** | **14** | **15** | **16** | **17** | **18** | **19** | **20** | **21** | **22** | **23** | **24** | **25** | **26** | **27** | **28** | **29** | **30** | **31** | **32** | **33** |
| --- | --- | --- | --- | --- | --- | --- | --- | --- | --- | --- | --- | --- | --- | --- | --- | --- | --- | --- | --- | --- | --- | --- | --- | --- | --- | --- | --- | --- | --- | --- | --- | --- | --- |
| *Synodus* | 0 | 0 | - | 0 | 0 | 0 | 0 | 0 | 0 | 0 | 0 | 0 | 0 | 0 | 0 | 0 | 0 | 0 | 0 | 0 | 0 | 0 | 0 | 0 | 0 | 0 | 0 | 0 | 0 | 0 | 0 | 0 | 0 |
| *Gymnoscopelus* | 0 | 0 | - | 1 | 0 | 1 | 0 | 0 | 0 | 1 | 0 | 0 | 0 | 0 | 0 | 0 | 0 | 0 | 0 | 0 | 0 | 0 | 0 | 1 | 0 | 0 | 0 | 0 | 0 | 0 | 0 | 0 | 0 |
| †*Ctenothrissa* | 0 | 0 | - | 0 | 0 | 0 | 0 | 0 | 0 | 0 | 0 | 0 | ? | 0 | 0 | 0 | 1 | 0 | 0 | 0 | 0 | 0 | 0 | 1 | ? | ? | 0 | ? | 0 | 0 | 0 | 0 | 1 |
| †*Pycnosteroides* | 0 | ? | ? | 0 | 0 | 1 | 1 | ? | 0 | 0 | 0 | ? | ? | 0 | 0 | ? | 1 | 0 | ? | 0 | ? | 0 | 0 | 1 | ? | 1 | 0 | ? | 0 | ? | 1 | 0 | 2/3 |
| †*'Aipichthys'* | 0 | 0 | - | 1 | 0 | 1 | 1 | ? | 0 | 0 | 0 | 0 | 1 | 0 | 0 | ? | 1 | 0 | 0 | 0 | ? | 0 | 0 | 1 | ? | 0 | 0 | ? | 0 | ? | 0 | 1 | 2 |
| †*Aipichthyoides* | 0 | 0 | - | 0 | 0 | 1 | 1 | ? | 1 | 0 | 0 | ? | 1 | 1 | 0 | ? | 1 | 0 | ? | ? | ? | 0 | 0 | 1 | ? | ? | 0 | ? | 0 | ? | 0 | 1 | 2 |
| *Velifer* | 1 | 0 | - | 1 | 1 | 1 | 1 | 1 | 1 | 0 | 0 | 0 | 1 | 1 | 1 | 0 | 1 | 0 | 1 | 1 | 0 | 1 | 0 | 1 | 3 | 0 | 1 | 1 | 0 | 0 | 1 | 1 | 3 |
| *Lampris* | 1 | 0 | - | 1 | 1 | 1 | 1 | 1 | 1 | 0 | 0 | 0 | 1 | 1 | 1 | 0 | 1 | 0 | 1 | 1 | 0 | 1 | 0 | 1 | 0 | 2 | 1 | 1 | 0 | 0 | 2 | 1 | 3 |
| *Regalecus* | 1 | 0 | - | 1 | 1 | 1 | 1 | 1 | 1 | 0 | 0 | - | 1 | - | 1 | 0 | 1 | 0 | 1 | 1 | 0 | 1 | 0 | 1 | ? | ? | ? | 1 | 0 | 1 | 3 | - | 3 |
| *Stylephorus* | 1 | 0 | - | 1 | 1 | 1 | 1 | ? | 1 | 1 | 1 | - | 1 | - | 0 | 1 | 1 | 1 | - | 1 | 0 | 0 | 0 | 1 | ? | ? | ? | - | 1 | 1 | 3 | - | 0 |
| *Polymixia* | 0 | 1 | 0 | 0 | 0 | 1 | 0 | 1 | 0 | 0 | 0 | 0 | 1 | 0 | 0 | 1 | 0 | 0 | 0 | 0 | 0 | 0 | 1 | 1 | 0 | 1 | 0 | 1 | 0 | 0 | 0 | 0 | 0 |
| †*Omosomopsis* | 0 | 1 | 0 | 1 | 0 | 1 | 1 | ? | ? | 0 | 1 | ? | 1 | 0 | 0 | ? | 1 | 0 | ? | 0 | ? | ? | 1 | 1 | ? | 1 | 0 | ? | 0 | ? | 0 | 0 | 1 |
| †*Sphenocephalus* | 0 | 1 | 1 | 1 | 0 | 1 | 0 | ? | ? | ? | ? | ? | 1 | 0 | 0 | 1 | 1 | 0 | ? | 0 | 0 | 0 | 0 | 1 | ? | ? | ? | 1 | 0 | 0 | 2 | 0 | 0/1 |
| *Aphredoderus* | 0 | 0 | - | 1 | 1 | 1 | 1 | 0 | 0 | 1 | 1 | 1 | 1 | 0 | 0 | 1 | 0 | 0 | 0 | 0 | 1 | 0 | 0 | 1 | 0 | 2 | 1 | 1 | 0 | 0 | 2 | 0 | 1 |
| *Percopsis* | 0 | 0 | - | 1 | 1 | 1 | 0 | 0 | 0 | 1 | 1 | 1 | 1 | 0 | 0 | 1 | 0 | 0 | 0 | 0 | 1 | 0 | 0 | 1 | 0 | 2 | 1 | 1 | 0 | 0 | 2 | 0 | 0 |
| *Bregmaceros* | 0 | 1 | 1 | 1 | 1 | 1 | 1 | 1 | 0 | 1 | 1 | 1 | 1 | 0 | 0 | 1 | 1 | 0 | ? | 1 | 0 | 0 | 0 | 1 | ? | ? | ? | ? | 1 | 1 | 3 | - | 1/2 |
| *Merluccius* | 0 | 1 | 1 | 1 | 1 | 1 | 1 | 1 | 0 | 1 | 1 | 1 | 1 | 0 | 0 | 1 | 1 | 0 | 0 | 1 | 1 | 0 | 0 | 1 | 0 | 2 | 1 | 1 | 1 | 1 | 3 | - | 1 |
| *Halobatrachus* | 1 | 1 | 0 | 1 | 1 | 1 | 1 | 1 | - | 1 | 1 | 0 | 1 | 0 | 0 | 1 | 0 | 0 | 0 | 0 | 1 | 0 | 0 | 1 | 1 | 2 | 1 | 1 | 1 | 0 | 3 | - | 1 |
| *Brotula* | 0 | 1 | 1 | 1 | 0 | 1 | 1 | 1 | 0 | 1 | 0 | 0 | 1 | 0 | 0 | 1 | 0 | 0 | 0 | 0 | 1 | 0 | 0 | 1 | 1 | 2 | 1 | 1 | 0 | 1 | 3 | 0 | 1 |
| *Cyttus* | 1 | 1 | 1 | 1 | 1 | 1 | 1 | 0 | 0 | 1 | 0 | 0 | 2 | 0 | 0 | 1 | 1 | 1 | 0 | 1 | 0 | 0 | 0 | 1 | 0 | ? | 1 | ? | 1 | 1 | 2 | - | 2 |
| *Zeus* | 1 | 1 | 1 | 1 | 1 | 1 | 1 | 0 | 0 | 1 | 1 | 0 | 2 | 0 | 0 | 1 | 1 | 1 | 0 | 1 | 0 | 0 | 0 | 1 | 2 | 2 | 1 | 1 | 1 | 1 | 3 | - | 2 |
| *Hoplostethus* | 0 | 1 | 1 | 0 | 0 | 1 | 0 | 1 | 0 | 0 | 0 | 0 | 1 | 0 | 0 | 1 | 0 | 0 | 0 | 0 | 0 | 0 | 0 | 1 | 1 | ? | 1 | 1 | 0 | 0 | 1 | 0 | 1 |
| *Sargocentron* | 0 | 1 | 0 | 0 | 0 | 1 | 0 | 1 | 0 | 0 | 0 | 0 | 1 | 0 | 0 | 1 | 0 | 0 | 0 | 0 | 0 | 0 | 0 | 1 | 1 | 2 | 1 | 1 | 0 | 0 | 0 | 0 | 2 |
| †*Stichocentrus* | 0 | 1 | ? | 0 | 0 | 1 | 0 | ? | 0 | 0 | 0 | ? | 0 | 0 | 0 | ? | 0 | 0 | ? | 0 | ? | 0 | 0 | 1 | ? | ? | ? | ? | 0 | ? | 1 | 0 | 2 |
| *Dicentrarchus* | 0 | 1 | 0 | 1 | 0 | 1 | 1 | 1 | 0 | 1 | 0 | 0 | 1 | 0 | 0 | 1 | 0 | 0 | 0 | 0 | 1 | 0 | 0 | 1 | 1 | 2 | 1 | 1 | 0 | 0 | 0 | 0 | 1 |
| *Lates* | 0 | 1 | 0 | 1 | 0 | 1 | 1 | 1 | 0 | 1 | 0 | 0 | 1 | 0 | 0 | 1 | 0 | 0 | 0 | 0 | 1 | 0 | 0 | 1 | 1 | ? | ? | 1 | 0 | 0 | 0 | 0 | 1 |
| **†*Spinocaudichthys*** | ? | ? | ? | ? | ? | ? | ? | ? | ? | ? | ? | ? | ? | 0 | ? | ? | ? | ? | ? | ? | ? | 0 | ? | ? | ? | 0/1 | 0 | ? | 0 | ? | 3 | - | 0 |

|  | **34** | **35** | **36** | **37** | **38** | **39** | **40** | **41** | **42** | **43** | **44** | **45** | **46** | **47** | **48** | **49** | **50** | **51** | **52** | **53** | **54** | **55** | **56** | **57** | **58** | **59** | **60** | **61** | **62** | **63** | **64** | **65** | **66** |
| --- | --- | --- | --- | --- | --- | --- | --- | --- | --- | --- | --- | --- | --- | --- | --- | --- | --- | --- | --- | --- | --- | --- | --- | --- | --- | --- | --- | --- | --- | --- | --- | --- | --- |
| *Synodus* | 0 | 0 | - | 0 | 0 | 0 | 0 | 0 | 0 | 0 | 0 | 0 | 1 | 0 | 0 | 1 | 0 | 0 | 0 | 0 | 0 | 0 | 0 | 0 | 0 | 0 | 0 | 0 | 0 | 0 | 0 | 0 | - |
| *Gymnoscopelus* | 0 | 0 | - | 0 | 0 | 0 | 0 | 0 | 1 | 1 | 0 | ? | 0 | 0 | 0 | 0 | 1 | 0 | 0 | 0 | 0 | 0 | 0 | 0 | 0 | 0 | 0 | 0 | 0 | 0 | 0 | 0 | - |
| †*Ctenothrissa* | 0 | 0 | - | 0 | 0 | 1 | 0 | 0 | 0 | 0 | 0 | 0 | 0 | 0 | 0 | 0 | 0 | 0 | 0 | 0 | 0 | 0 | 0 | 1 | 2 | ? | ? | ? | ? | ? | 0 | 0 | - |
| †*Pycnosteroides* | 0 | 1 | ? | 0 | 0 | 1 | 1 | 0 | 0 | 0 | 1 | 0 | 0 | 0 | 0 | 0 | 1 | 1 | 0 | 0 | 0 | 0 | 0 | 1 | 2 | ? | ? | ? | ? | ? | 0 | 1 | ? |
| †*'Aipichthys'* | 0 | 1 | ? | 0 | 1 | 1 | 1 | 0 | 0 | 0 | 0 | 0 | 0 | 0 | 0 | 0 | 1 | 0 | 0 | 0 | 0 | 0 | 0 | 1 | 2 | ? | ? | ? | 0 | ? | 0 | 0 | - |
| †*Aipichthyoides* | 0 | 1 | ? | 0 | 1 | 1 | 1 | 0 | 0 | 0 | 0 | 0 | 1 | 0 | 0 | 1 | 1 | 0 | 0 | 0 | 0 | 0 | 0 | 1 | 2 | ? | ? | ? | ? | ? | 0 | 0 | - |
| *Velifer* | 0 | 1 | ? | 0 | 1 | 1 | 1 | 0 | 1 | 0 | 0 | 0 | 0 | 0 | 0 | 0 | 1 | 0 | 0 | 1 | 0 | 0 | 0 | 1 | 2 | 0 | 0 | 1 | 0 | 0 | 0 | 0 | - |
| *Lampris* | 0 | 0 | - | 0 | 1 | 1 | 0 | 0 | 1 | 0 | 0 | 0 | 1 | 0 | 0 | 1 | 1 | 0 | 0 | 1 | 1 | 1 | 0 | 1 | 2 | 0 | 0 | 1 | 0 | 1 | 0 | 0 | - |
| *Regalecus* | 1 | 0 | - | 0 | 1 | 1 | - | 1 | 1 | 0 | - | - | 3 | 0 | 0 | 1 | - | 2 | - | 1 | 1 | 1 | 0 | 1 | 2 | 0 | ? | 1 | 0 | 1 | 1 | 0 | - |
| *Stylephorus* | 1 | 0 | - | 0 | 1 | 1 | 0 | 1 | 1 | 0 | - | - | 3 | 0 | 1 | 1 | - | 2 | 0 | 1 | 1 | 0 | 1 | 1 | 1 | 0 | ? | ? | ? | 1 | 1 | 0 | - |
| *Polymixia* | 0 | 1 | 0 | 0 | 0 | 1 | 1 | 0 | 0 | 0 | 1 | 0 | 0 | 0 | 0 | 0 | 0 | 1 | 0 | 0 | 0 | 0 | 0 | 1 | 0 | 0 | 0 | 0 | 1 | 0 | 0 | 0 | - |
| †*Omosomopsis* | 0 | 1 | 0 | 0 | 0 | 1 | 1 | 0 | 0 | 0 | 1 | 0 | 1 | 1 | 0 | 1 | 0 | 1 | 0 | 0 | 0 | 0 | 0 | 1 | 0 | ? | ? | ? | ? | ? | 0 | 0 | - |
| †*Sphenocephalus* | 0 | 1 | ? | 0 | 0 | 0 | 1 | 0 | 0 | 0 | 1 | 0 | 1 | 0 | 0 | 0 | 0 | 1 | 0 | 0 | 0 | 0 | 0 | 1 | 0 | 0 | 0 | 0 | 1 | ? | 0 | 1 | 0 |
| *Aphredoderus* | 0 | 1 | 0 | 0 | 0 | 1 | 1 | 0 | 1 | 0 | 1 | 0 | 1 | 0 | 1 | 0 | 0 | 1 | 0 | 1 | 0 | 0 | 0 | 1 | 0 | 0 | 0 | 0 | 1 | 0 | 0 | 0 | - |
| *Percopsis* | 0 | 1 | 0 | 0 | 0 | 0 | 1 | 0 | 1 | 0 | 1 | 0 | 1 | 0 | 0 | 0 | 0 | 1 | 0 | 1 | 0 | 0 | 0 | 0 | 0 | 0 | 0 | 0 | 1 | 0 | 0 | 0 | - |
| *Bregmaceros* | 1 | 0 | - | 0 | 1 | 1 | 0 | 0 | 1 | 0 | 1 | 1 | 1 | 1 | 1 | 1 | 0 | 2 | 1 | 1 | 0 | 1 | 0 | 1 | 1 | ? | 0 | ? | ? | ? | 0 | 0 | - |
| *Merluccius* | 0 | 0 | - | 0 | 0 | 1 | 0 | 0 | 1 | 0 | 1 | 1 | 1 | 1 | 1 | 1 | 0 | 2 | 1 | 1 | 0 | 0 | 0 | 1 | 1 | 0 | ? | 0 | 0 | ? | 0 | 0 | - |
| *Halobatrachus* | 0 | 1 | - | 1 | 0 | 1 | 0 | 0 | 1 | 0 | 1 | - | 1 | 0 | 0 | 1 | 0 | 2 | 0 | 1 | 0 | 0 | 0 | 1 | 1 | 1 | - | 0 | 0 | 1 | 1 | 1 | 1 |
| *Brotula* | 0 | 0 | - | 0 | 0 | 1 | 0 | 0 | 1 | 0 | 1 | - | 1 | 0 | 1 | 1 | 0 | 2 | 0 | 0 | 0 | 0 | 0 | 1 | 3 | ? | - | - | - | 1 | 1 | 1 | 1 |
| *Cyttus* | 0 | 1 | 0 | 1 | 0 | 1 | 1 | 1 | 1 | 1 | 1 | 1 | 1 | 0 | 1 | 1 | 0 | 2 | 0 | 1 | 0 | 0 | 1 | 1 | 1 | 0 | 0 | 1 | 0 | 1 | 0 | 1 | 0 |
| *Zeus* | 0 | 1 | 0 | 1 | 0 | 1 | 1 | 1 | 1 | 1 | 1 | 1 | 2 | 1 | 1 | 1 | 0 | 2 | 0 | 1 | 0 | 0 | 1 | 1 | 1 | 0 | 0 | 1 | 0 | 1 | 0 | 1 | 0 |
| *Hoplostethus* | 0 | 1 | 1 | 0 | 0 | 1 | 1 | 0 | 0 | 0 | 0 | 0 | 0 | 0 | 0 | 0 | 0 | 0 | 0 | 0 | 0 | 0 | 0 | 1 | 1 | 1 | 0 | 1 | 1 | 0 | 0 | 1 | 1 |
| *Sargocentron* | 0 | 1 | 1 | 1 | 0 | 1 | 1 | 0 | 1 | 1 | 0 | 1 | 0 | 0 | 0 | 1 | 0 | 0 | 0 | 0 | 0 | 0 | 0 | 1 | 1 | 1 | 0 | 1 | 0 | 0 | 0 | 1 | 1 |
| †*Stichocentrus* | 0 | 1 | ? | 0 | 0 | 1 | 1 | 0 | 0 | 0 | 0 | 0/1 | 0 | 0 | 0 | ? | 0 | 0 | 0 | 0 | 0 | 0 | 0 | 1 | 1 | ? | ? | ? | ? | ? | 0 | 1 | ? |
| *Dicentrarchus* | 0 | 1 | 1 | 1 | 0 | 1 | 1 | 0 | 1 | 1 | 0 | 0 | 0 | 0 | 0 | 1 | 0 | 2 | 0 | 0 | 0 | 0 | 0 | 1 | 1 | 1 | 1 | 1 | 0 | 1 | 1 | 1 | 1 |
| *Lates* | 0 | 1 | 1 | 1 | 0 | 1 | 1 | 0 | 1 | 1 | 0 | 0 | 1 | 0 | 0 | 1 | 0 | 2 | 0 | 0 | 0 | 0 | 0 | 1 | 1 | 1 | 1 | 1 | 0 | 1 | 1 | 1 | 1 |
| **†*Spinocaudichthys*** | 0 | 1 | ? | 0 | 0 | 1 | 1 | 0 | 0 | 0 | 1 | 0 | 1 | 0 | ? | 0 | 0 | 0 | ? | ? | ? | 0 | 0 | 0 | 0 | 0 | ? | ? | ? | ? | 0 | 0 | - |
